# Nocebo effects in systemic therapies for adult plaque psoriasis: A systematic review and meta-analysis

**DOI:** 10.3389/fmed.2024.1373520

**Published:** 2024-03-27

**Authors:** Bryan Ma, Ye-Jean Park, Kirk Barber, P. Régine Mydlarski

**Affiliations:** ^1^Division of Dermatology, Department of Medicine, Cumming School of Medicine, University of Calgary, Calgary, AB, Canada; ^2^Temerty Faculty of Medicine, University of Toronto, Toronto, ON, Canada

**Keywords:** psoriasis, systemic therapy, nocebo effect, randomized controlled trial, biologic

## Abstract

**Introduction:**

The nocebo effect is defined as adverse outcomes secondary to negative patient expectations rather than the pharmacologic activity of an intervention. Nocebo effects can reduce treatment adherence and/or persistence. Therefore, nocebo effects in psoriasis need to be defined.

**Methods:**

A Cochrane systematic review was updated with a search of MEDLINE, Embase, and the CENTRAL Register of Controlled Trials for phase II - IV RCTs comparing systemic therapy versus placebo for patients with moderate-to-severe plaque psoriasis. Estimates were pooled using a random effects model, and heterogeneity was evaluated using the *I*^2^ statistic. The primary outcome was the pooled proportion of any adverse event (AE) and corresponding risk difference (RD) in patients randomized to placebo versus systemic therapy.

**Results:**

A total of 103 unique trials were identified enrolling 43,189 patients. The overall pooled AE rate in patients randomized to systemic therapy was 57.1% [95% CI: 54.7–59.5%] compared to 49.8% [95% CI: 47.1–52.4%] for placebo [RD 6.7% (95% CI: 4.6–8.9%), *p* < 0.00001, *I*^2^ = 75%]. Both biologic and non-biologic systemic therapy groups had a higher proportion of infectious AEs compared to placebo. No statistically significant RD in serious AEs or AEs leading to discontinuation was identified between systemic therapy and placebo groups.

**Discussion:**

Half of patients exposed to inert placebo in clinical trials of systemic psoriasis therapies experienced AEs, which may be explained by nocebo effects. These findings have important implications when counseling patients and designing future studies.

## Introduction

Psoriasis is a multisystem, inflammatory skin disease associated with substantial morbidity and mortality (1–3). It is a chronic skin disorder that results in disfigurement, stigmatization, and disability, negatively impacting patient quality of life (4, 5). Further, it is linked to systemic conditions, such as cardiovascular disease, metabolic syndrome, inflammatory bowel disease, psoriatic arthritis, and depression (6, 7). An estimated 2% of the global population has psoriasis, of which 15–20% have severe disease requiring systemic therapy (8).

Systemic treatment options for severe psoriasis include retinoids, traditional immunosuppressants (such as methotrexate or cyclosporine), biologics, and oral small molecules (9). Over the past two decades, novel therapeutic agents, such as biologic therapies targeting TNF-α, IL-12/23, IL-17, and IL-23, have revolutionized psoriasis care such that near-total or total skin clearance has become the gold standard outcome measure used to assess treatment efficacy. However, these agents may be associated with side effects that negatively impact patient treatment adherence and/or persistence. In many instances, direct attribution and assessment of adverse event (AEs) causality can be difficult.

The nocebo effect is a well-established phenomenon defined as the occurrence of undesirable side effects secondary to negative patient expectations as opposed to the pharmacologic activity of an intervention (10–13). For example, Napadow et al. (14) previously demonstrated that patients with atopic dermatitis who anticipated exposure to an allergen reported increased itch with a control saline prick compared to those without similar preconceptions. The nocebo effect has important implications for both research and clinical care by limiting the accurate identification of treatment-emergent AEs in randomized controlled trials (RCTs), thereby increasing treatment-unrelated AEs in intervention arms, placebo arms, or both; resulting in the premature discontinuation of appropriate therapy, leading to increased disease burden and accumulation of disease-specific complications; and negatively influencing the patient-provider therapeutic relationship, reducing patient trust in selected medication options and impacting the provider’s approach to medication counseling.

To date, nocebo effects in psoriasis have not been thoroughly studied. Therefore, we performed a systematic review and meta-analysis of placebo-controlled RCTs of systemic therapies for moderate-to-severe plaque psoriasis with two objectives: to estimate the pooled proportion of patients randomized to placebo who experienced AEs, serious AEs (SAEs), AEs resulting in treatment discontinuation, infections, and injection- or infusion-related AEs; and to characterize the risk differences (RDs) in these outcomes between patients randomized to investigational product versus placebo, stratified by treatment class. Topical therapies were excluded from this review and meta-analysis to provide focus and depth on the exciting and rapidly growing market of systemic psoriasis treatments.

## Materials and methods

The Preferred Reporting Items for Systematic Reviews and Meta-Analyses (PRISMA) guidelines was employed (15).

### Search strategy

A living systematic review and network meta-analysis by the Cochrane Library has compiled phase II–IV RCTs of systemic therapies in adult patients with moderate-to-severe plaque psoriasis through to October 2021 (9). Eligible studies from this living review were included for analysis. The data was supplemented by searching Embase (Ovid), MEDLINE (Ovid), and the Cochrane CENTRAL Register of Controlled Trials up to January 1st, 2023. The full search strategy is outlined in Supplementary Table 1 and includes terms to capture psoriasis, systemic therapy, and placebo-controlled trials. References of relevant publications were also screened, and only studies published in the English language were included.

### Study selection

Studies were included for analysis using the following criteria: placebo-controlled phase II, III, or IV induction or maintenance clinical trials of patients aged ≥18 with moderate-to-severe plaque psoriasis; evaluation of conventional systemic anti-psoriatic agent [defined as methotrexate, cyclosporine, oral retinoid, fumaric acid ester, biologic, and/or oral small molecule (apremilast or deucravacitinib)] versus placebo; and published frequency and nature of AEs (including any AE, SAE, AE requiring treatment discontinuation, infections, and/or injection- or infusion-related AE) in both treatment and placebo groups. Phase I clinical trials were excluded given substantial methodological differences compared to phase II to IV studies.

All citations were independently reviewed by two separate investigators (BM and Y-JP) using the above predefined inclusion criteria. Studies were screened by title and abstract followed by full text review. Disagreements were settled by a third author (PM). All screening was performed using Covidence Systematic Review software (Veritas Health Innovation, Melbourne, Australia).

### Outcomes and data extraction

The primary outcome was the pooled proportion of patients experiencing any AE in the placebo arm, and associated RD between systemic therapy and placebo. Secondary outcomes included pooled RDs in SAEs, AEs requiring treatment discontinuation, infections, and infusion- or injection-related AEs between treatment and placebo groups. All AEs were defined and reported by the original study authors. For trials testing multiple interventions, the proportion of patients with each outcome was pooled by treatment class. Multiple doses of systemic therapy were pooled if applicable. Data only over the initial placebo-controlled portion of trials were included.

Trial features that were extracted included: study design and setting (phase, number of centers, duration of follow-up); psoriasis severity criteria; number of patients randomized to systemic therapy and placebo; and incidence and nature of AEs in both intervention and treatment arms. The Cochrane Collaboration Risk of Bias tool version 2.0 was used to assess the methodological quality of included trials (16).

### Statistical analysis

The proportion of patients experiencing the primary and secondary outcomes in each of the placebo and active treatment arms were pooled using a random effects model to account for between- and within-study heterogeneity. The Freeman-Tukey double arcsine transformation was used to compute 95% confidence intervals (CI) using the score statistic and exact binomial method. The pooled RD between placebo and intervention arms stratified by medication class (biologic versus non-biologic) were calculated using a restricted maximum likelihood random effects model with 95% CIs. Statistical heterogeneity was quantified using Cochran’s Q and *I*^2^ statistics, and interpreted based on Cochrane recommendations (*I*^2^ = 30–60% representing moderate, 50–90% substantial, and 75–100% considerable heterogeneity). Univariate meta-regression was used to explore potential causes of heterogeneity using the variables of publication year, trial phase, multinational versus single country study, number of trial centers, and medication class. Publication bias was assessed using funnel plots and Egger’s test. All analyses were performed in Review Manager 5.4 and Stata 17.0 using the *metaprop* program (StataCorp LLC, College Station, TX).

## Results

### Search results and included studies

**FIGURE 1:**
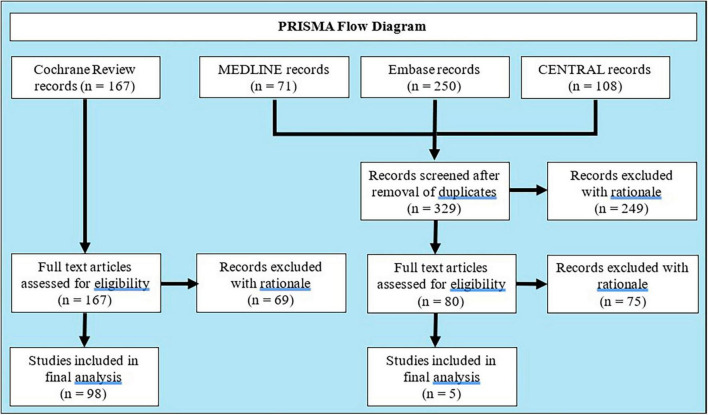
PRISMA flow diagram.

The final analysis included 103 unique RCTs representing 92 comparisons of biologic therapies and 38 comparisons of non-biologic treatments versus placebo (Figure 1 and Supplementary Appendix 1), enrolling a total of 30,249 patients randomized to systemic therapy (25,067 biologic, 82.9%) and 12,940 patients randomized to placebo. Ninety-six trials had initial placebo-controlled periods of 16 weeks or shorter. Four clinical trials comparing acitretin versus placebo were excluded from the analysis due to an inability to locate and confirm the primary outcomes (17–20). Any AE was reported in 96 comparisons (86 biologic and 10 non-biologic), SAEs in 110 comparisons (91 biologic and 19 non-biologic), AE leading to discontinuation of therapy in 107 comparisons (87 biologic and 20 non-biologic), and infectious AE in 101 comparisons (89 biologic and 12 non-biologic). A total of 52 comparisons of biologic agents reported either injection or infusion-related AEs. There was good agreement between reviewers on final studies for inclusion (Cohen’s Kappa 0.58, 93.8% agreement). Most trials were considered at low risk of randomization, missing data, and reporting bias (Supplementary Tables 2, 3).

### Risk difference in AEs

**FIGURE 2:**
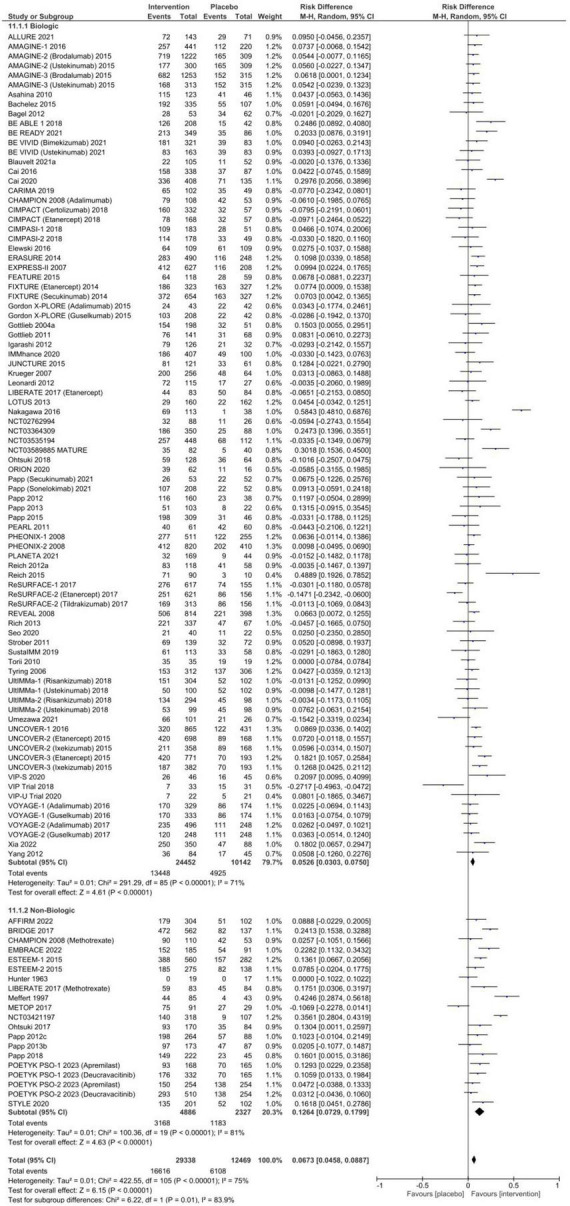
Pooled risk difference of any adverse event between patients treated with biologic or non-biologic therapy and placebo.

The pooled RD for any AE between systemic therapy and placebo is summarized in Figure 2 and Table 1. A total of 49.8% [95% CI: 47.1–52.4%] of patients randomized to placebo experienced an AE, compared to 57.1% [95% CI: 54.7–59.5%] in systemic therapy groups, resulting in a RD of 6.7% [95% CI: 4.6–8.9%, *p* < 0.00001] with considerable overall heterogeneity (*I*^2^ = 75%). This RD was observed in subgroup analyses for both biologic [RD 5.3% (95% CI: 3.0–7.5%), *p* < 0.00001, *I*^2^ = 71%] and non-biologic therapies [RD 12.6% (95% CI 7.3–18.0%), *p* < 0.00001, *I*^2^ = 81%] compared to placebo. There was no evidence of publication bias (Supplementary Figure 1, Egger *p*-value = 0.45).

**TABLE 1:** Pooled proportion of patients experiencing adverse events and risk difference between systemic therapy and placebo stratified by treatment class.

	Proportion% (95% CI)						Risk difference% (95% CI)		
	Intervention pooled	Placebo pooled	Biologic	Biologic placebo	Non-biologic	Non-biologic placebo	Biologic	Non-biologic	Pooled overall
Any AE	57.10 (54.66–59.52)	49.78 (47.12–52.44)	55.65 (53.14–58.15)	49.92 (47. 16–52.69)	63.02 (56.80–69.03)	48.75 (41.02–56.51)	**5.26 (3.03–7.50)**	**12.64 (7.29–17.99)**	**6.73 (4.58–8.87)**
Serious AE	1.79 (1.59–2.00)	1.42 (1.15–1.71)	1.82 (1.59–2.06)	1.29 (1.02–1.58)	1.70 (1.30–2.14)	1.87 (1.06–2.86)	**0.36 (0.06–0.67)**	–0.09 (–1.0–0.84)	0.28 (–0.01–0.58)
AE leading to discontinuation	2.17 (1.67–2.71)	1.90 (1.50–2.34)	1.40 (1.11–1.70)	1.49 (1.11–1.92)	6.47 (4.08–9.33)	3.61 (2.67–4.68)	0.12 (–0.17–0.40)	**2.85 (0.53–5.17)**	0.22 (0.53 – 5.17)
Infectious AE	20.27 (17.63–23.04)	15.57 (13.61–17.64)	20.09 (17.23–23.10)	15.56 (13.46–17.78)	21.51 (15.24–28.52)	15.69 (10.28–21.94)	**4.31 (2.83–5.79)**	**4.14 (1.50–6.78)**	**4.34 (2.99–5.69)**
Injection- or Infusion-related AE	NA	NA	4.92 (3.39–6.69)	1.69 (1.00–2.50)	NA	NA	**3.40 (1.91–4.89)**	NA	NA

AE, adverse event; CI, confidence interval; NA, not applicable. Bolded values are significantly different from 0 testing at a significance level of 5%.

**FIGURE 3:**
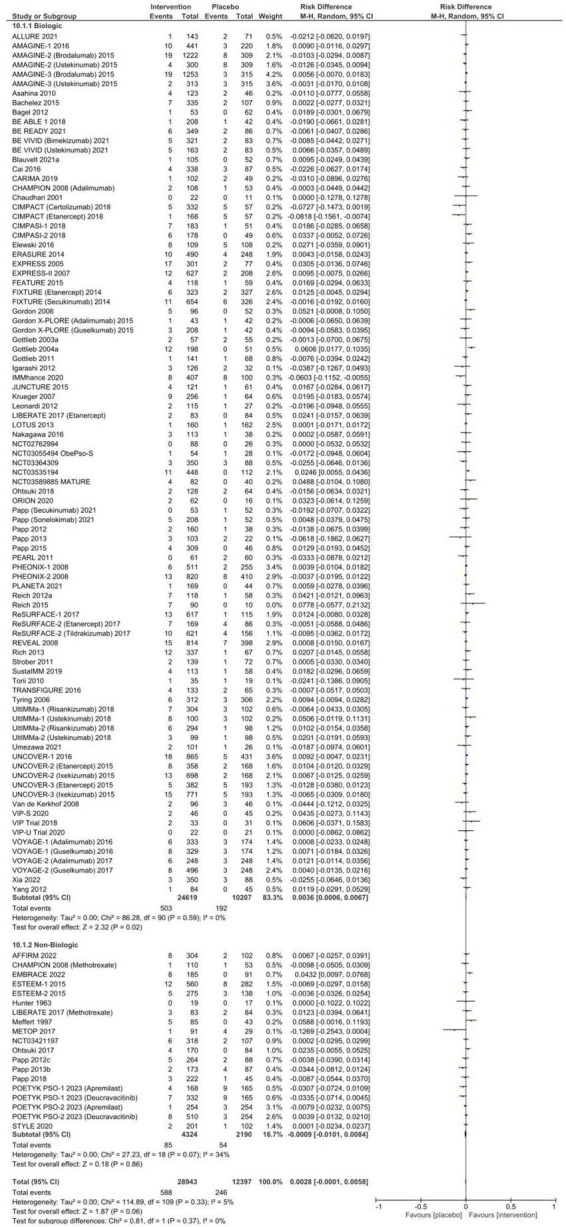
Pooled risk difference of serious adverse event between patients treated with biologic or non-biologic therapy and placebo.

**FIGURE 4:**
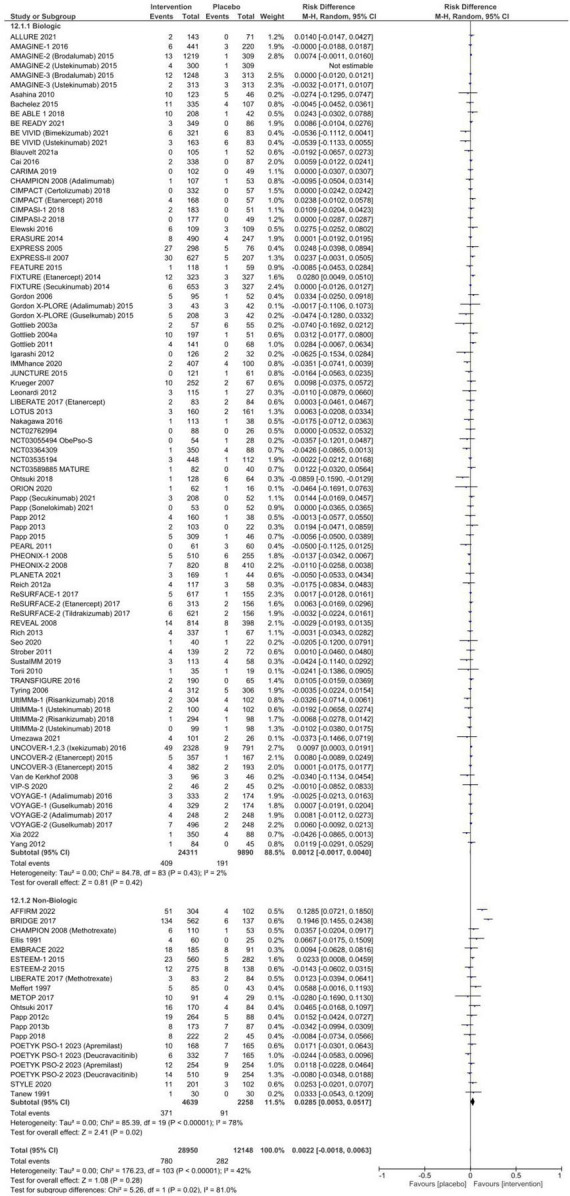
Pooled risk difference of adverse events leading to discontinuation of therapy between patients treated with biologic or non-biologic therapy and placebo.

The pooled proportion of patients receiving placebo who experienced an SAE was 1.4% [95% CI: 1.2–1.7%], and 1.9% [95% CI: 1.5–2.3%] of placebo patients discontinued therapy due to an AE. No statistically significant risk difference between patients who received systemic therapy and placebo for SAEs [RD 0.3% (95% CI 0.0–0.6%), *p* = 0.06, Figure 3] or AEs necessitating discontinuation of therapy [RD 0.2% (95% CI −0.2–0.6%), *p* = 0.28, Figure 4] was observed. In subgroup analysis, 6.5% (95% CI: 4.1–9.3%) of patients exposed to non-biologic agents experienced AEs requiring medication discontinuation compared to 3.6% [95% CI: 2.7–4.7%] of placebo patients (RD 2.9% [95% CI: 0.5–5.2%], *p* = 0.02) with considerable heterogeneity (*I*^2^ = 78%). Subclassification by year of publication, trial phase, multinational or single country, number of centers, and drug class in meta-regression resolved heterogeneity in SAEs (*I*^2^ = 14.75%), but only partially explained heterogeneity in AE leading to discontinuation (*I*^2^ = 49.50%).

**FIGURE 5:**
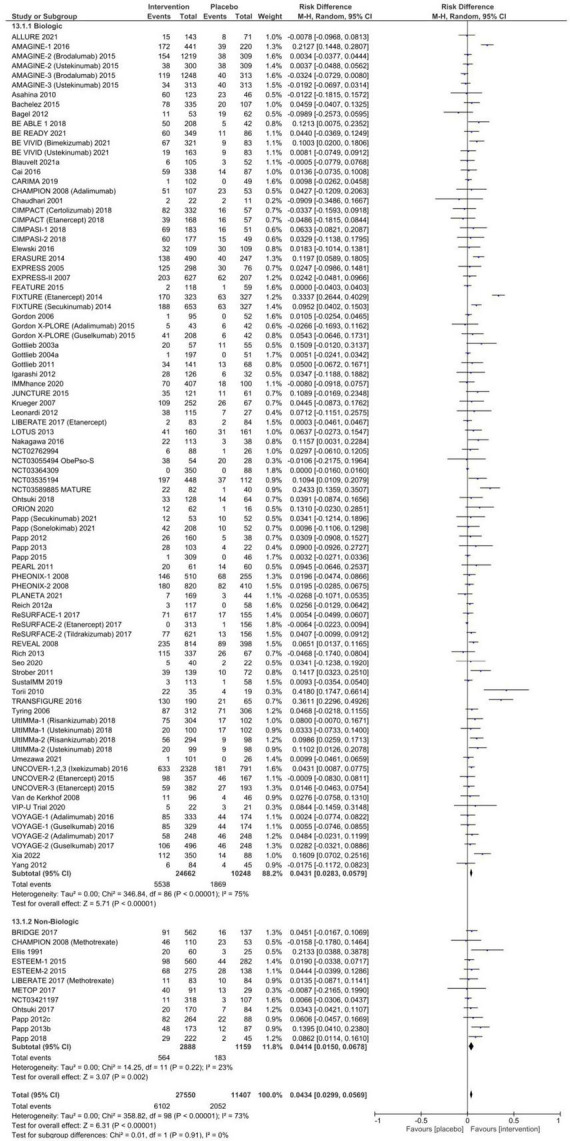
Pooled risk difference of infectious adverse events between patients treated with biologic or non-biologic therapy and placebo.

There was a higher risk of infections in patients receiving systemic therapy compared to placebo [RD 4.3% (95% CI 3.0–5.7%), *p* < 0.00001] that persisted in subgroup analyses for biologic agents [RD 4.3% (95% CI 2.8–5.8%), *p* < 0.00001, *I*^2^ = 75%, Figure 5] and non-biologic agents [RD 4.1% (95% CI: 1.5–6.8%), *p* = 0.002, *I*^2^ = 23%].

Injection- and infusion-related AEs are summarized in Supplementary Figure 2. There was a significant increase in the risk of injection or infusion-related AEs in the systemic therapy groups compared to placebo [RD 3.4% (95% CI 1.9–4.9%), *p* < 0.00001], although with significant heterogeneity (*I*^2^ = 89%). A total of 1.7% (95% CI: 1.0–2.5%) of placebo-treated patients experienced an injection or infusion-related AE.

## Discussion

In this systematic review and meta-analysis, we examined over 100 RCTs of systemic therapies for moderate-to-severe plaque psoriasis in more than 30,000 patients and identified several key findings. First, nearly 50% of patients receiving inert placebo experienced an AE. This high baseline rate of AEs may be partially explained by nocebo effects and has important implications for RCT design and for evaluating side effects in clinical care. Second, there were statistically significant but numerically low increased rates of all AEs and infections in trial participants receiving systemic therapies for psoriasis compared to placebo. However, we did not observe any significant difference in the proportion of placebo-treated compared to patients receiving systemic therapy who experienced SAEs or required treatment discontinuation, which should inform discussions with patients when starting systemic therapy.

### Research in context of existing literature

Randomized controlled trials (RCTs) of biologic and non-biologic agents for psoriasis have consistently identified non-specific medication-exposure related AEs (ex. nausea and headache) prone to nocebo effects (9). These effects have been well characterized in numerous drug classes, including HMG-CoA reductase inhibitors, (21) anti-depressants, (22) anti-epileptics, (23) and biologic therapies for other indications (24). However, there has been limited analysis of its role in systemic agents used for dermatologic indications. The pathogenesis of nocebo responses is complex and multifactorial, encompassing negative patient expectations secondary to perceived sensitivity to therapy, prior treatment experiences, and patient-provider therapeutic relationships (25–28). Furthermore, social conditioning from observed responses in others plays a key role, which is especially relevant given modern mass and social media-facilitated distribution of patient experiences with negative side effects and AEs (29). Therefore, patients with dermatologic conditions may be at high risk for nocebo effects because dermatologic diseases are generally highly visible, distressing, and frequently subject to both personal and peer judgment (i.e., stigmatization) that can predispose to negative interpretations of treatment-related events; and have a chronic, relapsing course such that patients may have negative expectations from failing multiple prior combination treatment regimens consisting of both systemic and topical agents.

### Impact of nocebo effects

Nocebo effects have important implications for RCT interpretation. First, high rates of AEs in patients randomized to placebo should be used to contextualize the overall safety profile of novel therapies in RCTs. Second, RCTs are essential for determining the efficacy of novel treatments. Still, they are generally under-powered for evaluating safety, especially for rare or serious AEs that require longer follow-up durations and greater patient exposure time, which is difficult to accommodate in most phase II and III induction and 1-year maintenance studies (30, 31). Third, nocebo effects in intervention groups may artificially increase rates of reported AEs and potentially mask the identification of true treatment-emergent AEs. Fourth, nocebo effects may lead to discontinuation of therapy and trial withdrawal, which can confound both evaluations of efficacy and safety if withdrawals occur differentially in treatment and placebo groups (32, 33). This has previously been observed in trials of patients switching from bio-originator to biosimilar TNF antagonists, despite numerous studies demonstrating bio-equivalence and non-inferiority (26). Reassuringly, we identified similar rates of treatment discontinuation between treatment and placebo due to AEs in psoriasis trials. Taken together, these findings highlight the importance of post-marketing drug registries, open-label trial extensions, and integrated safety analyses characterizing long-term safety outcomes.

Recognizing and minimizing nocebo effects in clinical practice may improve patient outcomes and enhance treatment persistence. While it is critical to ensure that all patients starting systemic therapy are informed of the risks and benefits of treatment, several strategies have been proposed to minimize nocebo effects. These include positive framing of side effect profiles, explicit disclosure of possible nocebo effects, standardized approaches to questioning for and measuring patient-reported AEs, and authorized concealment of limited disclosure of potentially rare or irrelevant AEs (28, 34–36). However, our findings need to be cautiously generalized to real-world practice given that clinical trials often select for an overall healthier patient population, whereas more comorbid patients may be using multiple concomitant therapies and be intrinsically at higher risk of AEs; and clinical constraints may alter the informed consent process and presentation of potential side effects compared to a controlled trial setting. The treatment context of an RCT itself may influence patient reporting of AEs because the processes of randomization, informed consent, and blinding have all been linked to nocebo effects (28, 37–39). For example, inclusion of possible gastrointestinal upset in written consent forms for unstable angina therapy was shown to increase the proportion of patients withdrawing from the study due to subjective, minor gastrointestinal symptoms by sixfold (39).

### Strengths and limitations

Our study has several important strengths. This systematic review and meta-analysis uniquely assesses the pooled proportion of patients experiencing AEs and associated RD between placebo and both biologic and non-biologic therapies in over 30,000 psoriasis patients. However, our study has some key limitations. First, alternative explanations for the high rate of AEs observed in placebo groups should also consider the accumulation of psoriatic complications from the natural history of progressive, untreated disease; potential misattribution of symptoms from related, comorbid psoriatic conditions such as psoriatic arthritis; and potential effects of concomitant topical or systemic therapies for either psoriasis or an associated condition. Second, there was significant heterogeneity between studies when assessing for any AE that was not fully explained in meta-regression. This may be a consequence of differences in defining and reporting AEs, patient characteristics (such as psoriatic involvement of special sites, concomitant psoriatic complications), and/or intervention differences between sub-therapy classes. For example, oral non-biologic medications, like cyclosporine and acitretin, have different mechanisms of action, resulting in potentially distinct side effect profiles.

In conclusion, we performed a comprehensive systematic review and meta-analysis of systemic therapies for moderate-to-severe plaque psoriasis. Nearly half of all patients randomized to placebo experienced AEs. Our evaluation reveals the necessity of considering nocebo effects to account for these findings. We did not identify any significant overall RD in either serious AE or AE leading to discontinuation of therapy between systemic therapy and placebo. These outcomes inform the interpretation of RCT data and influence clinician-patient communication.

## Data availability statement

The original contributions presented in this study are included in this article/Supplementary material, further inquiries can be directed to the corresponding author.

## Author contributions

BM: Writing – review & editing, Writing – original draft, Visualization, Validation, Software, Methodology, Investigation, Formal Analysis, Data curation, Conceptualization. Y-JP: Writing – review & editing, Writing – original draft, Methodology, Investigation, Data curation. KB: Writing – review & editing, Writing – original draft. PRM: Visualization, Validation, Supervision, Software, Resources, Project administration, Methodology, Investigation, Funding acquisition, Formal Analysis, Data curation, Conceptualization, Writing – review & editing, Writing – original draft.
