# Corrigendum: Nocebo effects in systemic therapies for adult plaque psoriasis: a systematic review and meta-analysis

**DOI:** 10.3389/fmed.2024.1439345

**Published:** 2024-06-27

**Authors:** Bryan Ma, Ye-Jean Park, Kirk Barber, P. Régine Mydlarski

**Affiliations:** ^1^Division of Dermatology, Department of Medicine, Cumming School of Medicine, University of Calgary, Calgary, AB, Canada; ^2^Temerty Faculty of Medicine, University of Toronto, Toronto, ON, Canada

**Keywords:** psoriasis, systemic therapy, nocebo effect, randomized controlled trial, biologic

In the published article, there was an error in Table 1 as published. In the row “Any AE” under the columns “Non-biologic” and “Non-biologic Placebo,” the numbers entered of 57.10 (54.66 – 59.52) and 49.78 (47.12 – 52.44), respectively, are an accidental transposition of these same numbers from the “Intervention Pooled” and “Placebo Pooled” columns. Instead, the numbers under row “Any AE” for column “Non-biologic” should be 63.02 (56.80 – 69.03) and for column “Non-biologic Placebo” should be 48.75 (41.02 – 56.51). The corrected Table 1 appears below.

**Table 1:** Pooled proportion of patients experiencing adverse events and risk difference between systemic therapy and placebo stratified by treatment class.

	**Proportion % (95% CI)**						**Risk difference % (95% CI)**		
	**Intervention pooled**	**Placebo pooled**	**Biologic**	**Biologic placebo**	**Non-biologic**	**Non-biologic placebo**	**Biologic**	**Non-biologic**	**Pooled overall**
Any AE	57.10 (54.66 – 59.52)	49.78 (47.12 – 52.44)	55.65 (53.14 – 58.15)	49.92 (47. 16 – 52.69)	63.02 (56.80 – 69.03)	48.75 (41.02 – 56.51)	**5.26 (3.03 – 7.50)**	**12.64 (7.29 – 17.99)**	**6.73 (4.58 – 8.87)**
Serious AE	1.79 (1.59 – 2.00)	1.42 (1.15 – 1.71)	1.82 (1.59 – 2.06)	1.29 (1.02 – 1.58)	1.70 (1.30 – 2.14)	1.87 (1.06 – 2.86)	**0.36 (0.06 – 0.67)**	−0.09 (-1.0 – 0.84)	0.28 (-0.01 – 0.58)
AE leading to discontinuation	2.17 (1.67 – 2.71)	1.90 (1.50 – 2.34)	1.40 (1.11 – 1.70)	1.49 (1.11 – 1.92)	6.47 (4.08 – 9.33)	3.61 (2.67 – 4.68)	0.12 (-0.17 – 0.40)	**2.85 (0.53 – 5.17)**	0.22 (0.53 – 5.17)
Infectious AE	20.27 (17.63 – 23.04)	15.57 (13.61 – 17.64)	20.09 (17.23 – 23.10)	15.56 (13.46 – 17.78)	21.51 (15.24 – 28.52)	15.69 (10.28 – 21.94)	**4.31 (2.83 – 5.79)**	**4.14 (1.50 – 6.78)**	**4.34 (2.99 – 5.69)**
Injection- or Infusion-related AE	NA	NA	4.92 (3.39 – 6.69)	1.69 (1.00 – 2.50)	NA	NA	**3.40 (1.91 – 4.89)**	NA	NA

AE, adverse event; CI, confidence interval; NA, not applicable.

Bolded values are significantly different from 0 testing at a significance level of 5%.

The authors apologize for this error and state that this does not change the scientific conclusions of the article in any way. The original article has been updated.

