# Supplementary material for: Nocebo effects in systemic therapies for adult plaque psoriasis: A systematic review and meta-analysis
Source: Front Med (Lausanne). 2024 Mar 27;11:1373520. doi: 10.3389/fmed.2024.1373520 (PMC11004429; doi:10.3389/fmed.2024.1373520)
Supplement: Supplementary file 1 [file Table_1.DOCX]

**TABLE OF CONTENTS**

**Supplementary Table 1: S**earch strategy

**Supplementary Table 2:** Cochrane Risk of Bias assessment of identified additional studies. **Supplementary Table 3:** Characteristics of identified additional studies.

**Supplementary Figure 1:** Funnel plot of pooled any adverse event.

**Supplementary Figure 2:** Pooled risk difference of infusion- or injection-related adverse event between patients treated with biologic therapy and placebo.

**Supplementary Appendix 1:** Bibliographies of included studies from Cochrane living systematic review and meta-analysis.

**Supplementary Table 1:** Search strategy

| **MEDLINE and Embase**  1 exp Psoriasis/  2 psoria*.tw,kf.  3 1 or 2  4 exp Administration, Oral/  5 oral*.tw,kf.  6 ("per os" or PO).tw,kf.  7 inject*.tw,kf.  8 systemic.tw,kf.  9 4 or 5 or 6 or 7 or 8  10 exp Retinoids/  11 retino*.tw,kf.  12 exp Acitretin/  13 acitretin.tw,kf.  14 soriatane.tw,kf.  15 exp Isotretinoin/  16 isotretinoin.tw,kf.  17 accutane.tw,kf.  18 exp Steroids/  19 steroid*.tw,kf.  20 corticosteroid*.tw,kf.  21 glucocorticoid.tw,kf.  22 exp Methotrexate/  23 Methotrexate*.tw,kf.  24 MTX.tw,kf.  25 Amethopterin*.tw,kf.  26 Mexate.tw,kf.  27 Reditrex.tw,kf.  28 Trexall.tw,kf.  29 Xatmep.tw,kf.  30 Otrexup.tw,kf.  31 Rasuvo.tw,kf.  32 c?closporin?.tw,kf.  33 exp Cyclosporine/  34 phosphodiesterase.tw,kf.  35 PDE4.tw,kf.  36 Apremilast.tw,kf.  37 Otezla.tw,kf.  38 exp Biological Products/  39 Biologic*.tw,kf.  40 exp Biosimilar Pharmaceuticals/  41 biosimilar.tw,kf.  42 exp Antibodies, Monoclonal/  43 monoclonal antibod*.tw,kf.  44 exp Tumor Necrosis Factor-alpha/  45 tumor necrosis factor alpha.tw,kf.  46 tumor necrosis factor receptor.tw,kf.  47 anti-TNF.tw,kf.  48 Anti tumor necrosis factor.tw,kf.  49 TNFi.tw,kf.  50 TNF inhibitor.tw,kf.  51 exp Infliximab/  52 infliximab.tw,kf.  53 remicade.tw,kf.  54 exp Etanercept/  55 etanercept.tw,kf.  56 Enbrel.tw,kf.  57 adalimumab.tw,kf.  58 exp Adalimumab/  59 humira.tw,kf.  60 exp Antirheumatic Agents/  61 golimumab.tw,kf.  62 simponi.tw,kf.  63 exp Certolizumab Pegol/  64 certolizumab.tw,kf.  65 Cimzia.tw,kf.  66 exp Interleukins/  67 interleukin*.tw,kf.  68 IL.tw,kf. 411517  69 IL-23.tw,kf.  70 IL-17.tw,kf.  71 IL-17A.tw,kf.  72 IL-12.tw,kf.  73 IL-39.tw,kf.  74 exp Ustekinumab/  75 Stelara.tw,kf.  76 ustekinumab.tw,kf.  77 Cosentyx.tw,kf.  78 secukinumab.tw,kf.  79 brodalumab.tw,kf.  80 Siliq.tw,kf.  81 ixekizumab.tw,kf.  82 Taltz.tw,kf.  83 tildrakizumab.tw,kf.  84 ilumya.tw,kf.  85 Risankizumab.tw,kf.  86 skyrizi.tw,kf.  87 guselkumab.tw,kf.  88 Tremfya.tw,kf.  89 bimekizumab.tw,kf.  90 Bimzelx.tw,kf.  91 exp Janus Kinases/  92 janus kinase*.tw,kf.  93 JAK?.tw,kf.  94 JAK inhibitor.tw,kf.  95 Janus kinase inhibitor.tw,kf.  96 tofacitinib.tw,kf.  97 Xeljanz.tw,kf.  98 baricitinib.tw,kf.  99 Olumiant.tw,kf.  100 deucravacitinib.tw,kf.  101 Sotyktu.tw,kf.  102 10 or 11 or 12 or 13 or 14 or 15 or 16 or 17 or 18 or 19 or 20 or 21 or 22 or 23 or 24 or 25 or 26 or 27 or 28 or 29 or 30 or 31 or 32 or 33 or 34 or 35 or 36 or 37 or 38 or 39 or 40 or 41 or 42 or 43 or 44 or 45 or 46 or 47 or 48 or 49 or 50 or 51 or 52 or 53 or 54 or 55 or 56 or 57 or 58 or 59 or 60 or 61 or 62 or 63 or 64 or 65 or 66 or 67 or 68 or 69 or 70 or 71 or 72 or 73 or 74 or 75 or 76 or 77 or 78 or 79 or 80 or 81 or 82 or 83 or 84 or 85 or 86 or 87 or 88 or 89 or 90 or 91 or 92 or 93 or 94 or 95 or 96 or 97 or 98 or 99 or 100 or 101  103 9 and 102  104 exp Phototherapy/  105 phototherap*.tw,kf.  106 exp PUVA Therapy/  107 PUVA.tw,kf.  108 exp Ultraviolet Therapy/  109 exp Ficusin/  110 psoralen*.tw,kf.  111 exp Photochemotherapy/  112 photochemotherap*.tw,kf.  113 (BB-UVB or BBUVB).tw,kf.  114 (NB-UVB or NBUVB).tw,kf.  115 ((broad band or BB) adj1 (ultraviolet or UV or light or radiation)).tw,kf.  116 ((narrow band or NB) adj1 (ultraviolet or UV or light or radiation)).tw,kf.  117 Ultraviolet.tw,kf.  118 UV.tw,kf.  119 photodynamic therap*.tw,kf.  120 PDT.tw,kf.  121 104 or 105 or 106 or 107 or 108 or 109 or 110 or 111 or 112 or 113 or 114 or 115 or 116 or 117 or 118 or 119 or 120  122 103 or 121  123 3 and 122  124 limit 123 to (clinical study or clinical trial, all or controlled clinical trial or randomized controlled trial)  125 randomized controlled trial.pt.  126 placebo controlled trial.pt.  127 trial.tw.  128 placebo.tw,kf.  129 random*.tw,kf.  130 (Crossover or cross over or cross-over).tw,kf.  131 single-blind.tw,kf.  132 double-blind.tw,kf.  133 triple-blind.tw,kf.  134 blind.tw,kf.  135 allocat*.tw,kf.  136 125 or 126 or 127 or 128 or 129 or 130 or 131 or 132 or 133 or 134 or 135  137 124 and 136  138 limit 137 to humans  139 limit 138 to yr="2021 - 2023" | **CENTRAL**  #1 MeSH descriptor: [Psoriasis] explode all trees  #2 psoria*  #3 #1 or #2  #4 oral or per os or PO  #5 inject*  #6 systemic  #7 #4 or #5 or #6  #8 MeSH descriptor: [Retinoids] explode all trees  #9 retino*  #10 MeSH descriptor: [Acitretin] explode all trees  #11 soriatane  #12 MeSH descriptor: [Isotretinoin] explode all trees  #13 accutane  #14 MeSH descriptor: [Glucocorticoids] explode all trees  #15 MeSH descriptor: [Methotrexate] explode all trees  #16 MTX  #17 amethopterin*  #18 Mexate  #19 reditrex  #20 trexall  #21 xatmep  #22 otrexup  #23 rasuvo  #24 MeSH descriptor: [Cyclosporins] explode all trees  #25 MeSH descriptor: [Phosphodiesterase Inhibitors] explode all trees  #26 PDE4  #27 Apremilast  #28 Otezla  #29 MeSH descriptor: [Biological Products] explode all trees  #30 MeSH descriptor: [Biosimilar Pharmaceuticals] explode all trees  #31 MeSH descriptor: [Antibodies, Monoclonal] explode all trees  #32 MeSH descriptor: [Tumor Necrosis Factor Inhibitors] explode all trees  #33 MeSH descriptor: [Tumor Necrosis Factor-alpha] explode all trees  #34 MeSH descriptor: [Receptors, Tumor Necrosis Factor] explode all trees  #35 TNFi  #36 TNF inhibitor  #37 MeSH descriptor: [Infliximab] explode all trees  #38 remicade  #39 MeSH descriptor: [Etanercept] explode all trees  #40 enbrel  #41 MeSH descriptor: [Adalimumab] explode all trees  #42 humira  #43 MeSH descriptor: [Certolizumab Pegol] explode all trees  #44 certolizumab  #45 cimzia  #46 golimumab  #47 simponi  #48 MeSH descriptor: [Interleukins] explode all trees  #49 interleukin*  #50 IL  #51 IL-23  #52 IL-17  #53 IL-17A  #54 IL-12  #55 IL-39  #56 MeSH descriptor: [Ustekinumab] explode all trees  #57 stelara  #58 secukinumab  #59 cosentyx  #60 brodalumab  #61 siliq  #62 ixekizumab  #63 taltz  #64 tildrakizumab  #65 ilumya  #66 risankizumab  #67 skyrizi  #68 guselkumab  #69 tremfya  #70 bimekizumab  #71 bimzelx  #72 MeSH descriptor: [Janus Kinases] explode all trees  #73 MeSH descriptor: [Janus Kinase Inhibitors] explode all trees  #74 JAK?  #75 JAK inhibitor  #76 janus kinase inhibitor  #77 tofacitinib  #78 xeljanz  #79 baricitinib  #80 olumiant  #81 deucravacitinib  #82 sotyktu  #83 #7 or #8 or #9 or #10 or #11 or #12 or #13 or #14 or #15 or #16 or #17 or #18 or #19 or #20 or #21 or #22 or #23 or #24 or #25 or #26 or #27 or #28 or #29 or #30 or #31 or #32 or #33 or #34 or #35 or #36 or #37 or #38 or #39 or #40 or #41 or #42 or #43 or #44 or #45 or #46 or #47 or #48 or #49 or #50 or #51 or #52 or #53 or #54 or #55 or #56 or #57 or #58 or #59 or #60 or #61 or #62 or #63 or #64 or #65 or #66 or 67 or #68 or #69 or #70 or #71 or #72 or #73 or #74 or #75 or #76 or #77 or #78 or #79 or #80 or #81 or #82  #84 #3 and #83  #85 MeSH descriptor: [Phototherapy] explode all trees  #86 phototherap*  #87 MeSH descriptor: [PUVA Therapy] explode all trees  #88 MeSH descriptor: [Ultraviolet Therapy] explode all trees  #89 ficusin  #90 psoralen*  #91 MeSH descriptor: [Photochemotherapy] explode all trees  #92 photochemotherap*  #93 BB-UVB or BBUVB or NB-UVB or NBUVB  #94 (broad band or bb) adj1 (ultraviolet or UV or light or radiation)  #95 (narrow band or NB) adj1 (ultraviolet or UV or light or radiation)  #96 ultraviolet  #97 photodynamic therap*  #98 PDT  #99 #85 or #86 or #87 or #88 or #89 or #90 or #91 or #92 or #93 or #94 or #95 or #96 or #97 or #98  #100 #84 or #99  #101 #3 and #100  #102 #101 in Trials  #103 MeSH descriptor: [Randomized Controlled Trial] explode all trees  #104 placebo controlled trial  #105 trial  #106 placebo  #107 random*  #108 crossover or cross over or cross-over  #109 single-blind or double-blind or triple-blind or blind  #110 allocat*  #111 #103 or #104 or #105 or #106 or #107 or #108 or #109 or #110  #112 #102 and 111  #113 #112 with Publication Year from 2021 to 2023, in Trials |
| --- | --- |

| **Study ID** | **Experimental** | **Comparator** | **Outcome** | **D1** | **D2** | **D3** | **D4** | **D5** | **Overall** |  |  |  |
| --- | --- | --- | --- | --- | --- | --- | --- | --- | --- | --- | --- | --- |
| Xia 2022 | Ixekizumab | Placebo | PASI and sPGA |  |  |  |  |  |  |  |  | Low risk |
| AFFIRM 2022 | FAE | Placebo | PASI-75 and IgA 0/1 |  |  |  |  |  |  |  |  | Some concerns |
| EMBRACE 2022 | Apremilast | Placebo | DLQI |  |  |  |  |  |  |  |  | High risk |
| POETYK PsO-1 | Apremilast and Deucravacitinb | Placebo | PASI-75 and sPGA 0/1 |  |  |  |  |  |  |  |  |  |
| POETYK PSO-2 | Apremilast and Deucravacitinib | Placebo | PASI-75 and sPGA 0/1 |  |  |  |  |  |  |  | D1 | Randomisation process |
|  |  |  |  |  |  |  |  |  |  |  | D2 | Deviations from the intended interventions |
|  |  |  |  |  |  |  |  |  |  |  | D3 | Missing outcome data |
|  |  |  |  |  |  |  |  |  |  |  | D4 | Measurement of the outcome |
|  |  |  |  |  |  |  |  |  |  |  | D5 | Selection of the reported result |

**Supplementary Table 2:** Cochrane Risk of Bias assessment of identified additional studies.

**Supplementary Table 3:** Extracted characteristics of identified additional studies.

| Trial Identifier | Trial Phase | Trial Centres (n) | Intervention | Psoriasis severity | Primary outcome | Total patients (n) | Intervention patients (n) | Placebo patients (n) | Total Duration (weeks) |
| --- | --- | --- | --- | --- | --- | --- | --- | --- | --- |
| Xia 2022 | 3 | 17 | Ixekizumab | PASI ≥12, sPGA ≥3, ≥10% BSA | PASI | 438 | 350 | 88 | 60 |
| AFFIRM 2022 | 2b | 74 | Apremilast | PASI ≥12, BSA ≥10%, IGA≥3 | PASI | 406 | 304 | 102 | 24 |
| EMBRACE 2022 | 4 | NR | FAE | ≥1 special area, DLQI >10, PASI 3 – 10 | DLQI | 276 | 185 | 91 | 16 |
| POETYK PSO-1 2023a | 3 | 154 | Apremilast | PASI ≥12, BSA ≥10%, sPGA≥3 | PASI | 301 | 168 | 165 | 52 |
| POETYK PSO-1 2023d | 3 | 154 | Deucravacitinib | PASI ≥12, BSA ≥10%, sPGA≥3 | PASI | 497 | 332 | 165 | 52 |
| POETYK PSO-2 2023a | 3 | 191 | Apremilast | PASI ≥12, BSA ≥10%, sPGA≥3 | PASI | 301 | 168 | 165 | 52 |
| POETYK PSO-2 2023d | 3 | 191 | Deucravacitinib | PASI ≥12, BSA ≥10%, sPGA≥3 | PASI | 764 | 510 | 254 | 52 |

BSA, body surface area; DLQI, dermatology life quality index; FAE, fumaric acid ester; PASI, psoriasis area and severity index; sPGA, static Physician Global Assessment.


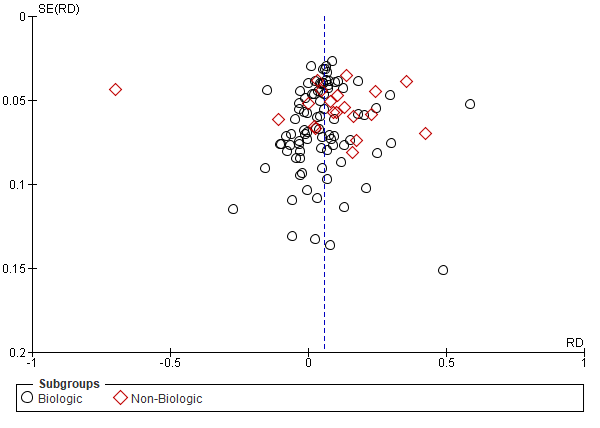


Egger’s p = 0.45 (biologic)

p = 0.39 (non-biologic)

**Supplementary Figure 1:** Funnel plot of pooled any adverse event.


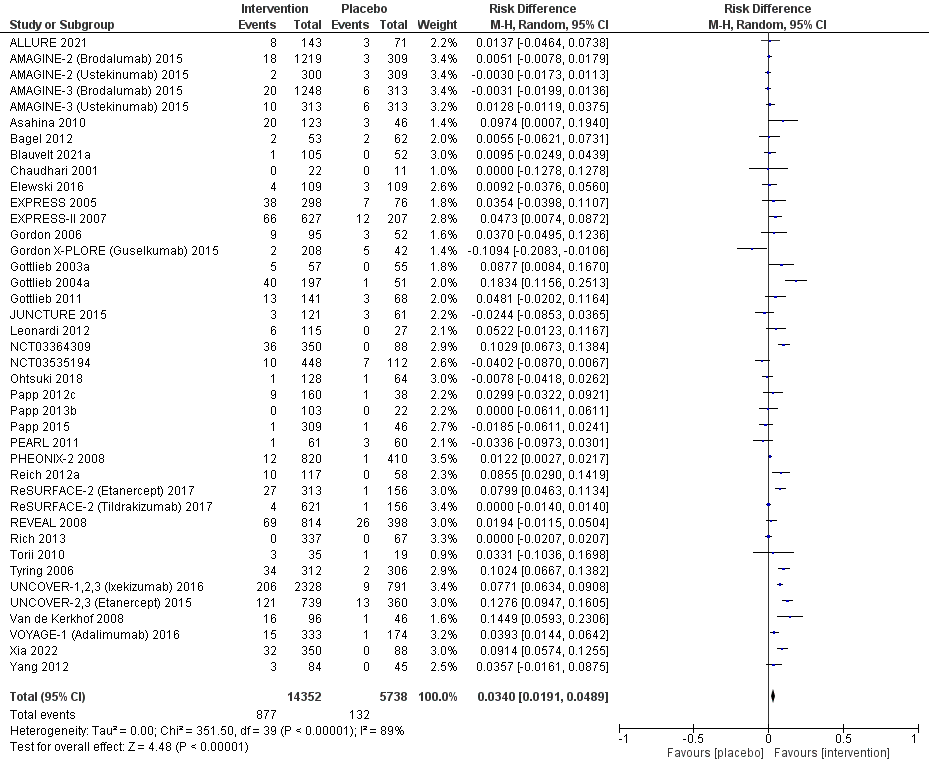


**Supplementary Figure 2:** Pooled risk difference of infusion- or injection-related adverse event between patients treated with biologic therapy and placebo.

**Supplementary Appendix 1:** Bibliographies of included studies from Cochrane living systematic review and meta-analysis.

**Acitretin**

1. Lowe NJ, Prystowsky JH, Bourget T, Edelstein J, Nychay S, Armstrong R. Acitretin plus UVB therapy for psoriasis. Comparisons with placebo plus UVB and acitretin alone. Journal of the American Academy of Dermatology 1991;24(4):591-4. [CENTRAL: CN-00075422] [PMID: 1827799]
2. Olsen EA, Weed WW, Meyer CJ, Cobo LM. A double-blind, placebo-controlled trial of acitretin for the treatment of psoriasis. Journal of the American Academy of Dermatology 1989;21(4 Pt 1):681-6. [CENTRAL: CN-00063370] [PMID: 2530251]
3. Tanew A, Guggenbichler A, Hönigsmann H, Geiger JM, Fritsch P. Photochemotherapy for severe psoriasis without or in combination with acitretin: a randomized, double blind comparison study. Journal of the American Academy of Dermatology 1991;25(4):682-4. [CENTRAL: CN-00612571] [PMID: 1838750]
4. Ye T, Ye J, Zhang C. The effects of acitretin on patients with psoriasis vulgaris. International Journal of Clinical and Experimental Medicine 2020;13(7):5068-75.

**Adalimumab**

1. Asahina A, Nakagawa H, Etoh T, Ohtsuki M, Adalimumab M04-688 Study Group. Adalimumab in Japanese patients with moderate to severe chronic plaque psoriasis: efficacy and safety results from a Phase II/III randomized controlled study. Journal of Dermatology 2010;37(4):299-310. [CENTRAL: CN-00762123] [PMID: 20507398]
2. Cai L, Gu J, Zheng J, Zheng M, Wang G, Xi LY, et al. Efficacy and safety of adalimumab in Chinese patients with moderate-to severe plaque psoriasis: results from a phase 3, randomized, placebo-controlled, double-blind study. Journal of the European Academy of Dermatology and Venereology 2016;31(1):89-95. [CENTRAL: CN-01248561] [PMID: 27504914]
3. Saurat JH, Stingl G, Dubertret L, Papp K, Langley RG, Ortonne JP, et al. Efficacy and safety results from the randomized controlled comparative study of adalimumab vs. methotrexate vs. placebo in patients with psoriasis (CHAMPION). British Journal of Dermatology 2008;158(3):558-66. [CENTRAL: CN-00628565] [PMID: 18047523]
4. Elewski BE, Rich PA, Okun MM, Papp K, Baker CS, Crowley JJ, et al. Adalimumab for nail psoriasis: efficacy and safety from the first 26 weeks of a phase-3, randomized, placebo-controlled trial. Journal of the European Academy of Dermatology and Venereology 2016;30(Suppl 6):65. [CENTRAL: CN-01786943] [EMBASE: 611235503]
5. Gordon KB, Langley RG, Leonardi C, Toth D, Menter MA, Kang S, et al. Clinical response to adalimumab treatment in patients with moderate to severe psoriasis: double-blind, randomized controlled trial and open-label extension study. Journal of the American Academy of Dermatology 2006;55(4):598-606. [CENTRAL: CN-00568251] [PMID: 17010738]
6. Gordon KB, Dufin KC, Bissonnette R, Prinz JC, Wasfi Y, Li S, et al. A phase 2 trial of guselkumab versus adalimumab for plaque psoriasis. New England Journal of Medicine 2015;373(2):136-44. [CENTRAL: CN-01076768] [PMID: 26154787]
7. Menter A, Augustin M, Signorovitch J, Yu AP, Wu EQ, Gupta SR, et al. The effect of adalimumab on reducing depression symptoms in patients with moderate to severe psoriasis: a randomized clinical trial. Journal of the American Academy of Dermatology 2010;62(5):812-8. [CENTRAL: CN-00743358] [PMID: 20219265]
8. Mehta NN, Shin DB, Joshi AA, Dey AK, Armstrong AW, Duffin KC, et al. Effect of 2 psoriasis treatments on vascular inflammation and novel inflammatory cardiovascular biomarkers: a randomized placebo-controlled trial. Circulation. Cardiovascular Imaging 2018;11(6):e007394. [CENTRAL: CN-01652064] [DOI: 10.1161/CIRCIMAGING.117.007394]
9. Blauvelt A, Papp KA, Griffiths CE, Randazzo B, Wasfi Y, Shen Y-K, et al. Efficacy and safety of guselkumab, an anti-interleukin-23 monoclonal antibody, compared with adalimumab for the continuous treatment of patients with moderate to severe psoriasis: results from the phase III, double-blinded, placebo- and active comparator-controlled VOYAGE 1 trial. Journal of the American Academy of Dermatology 2016;76(3):405-17. [CENTRAL: CN-01341398] [PMID: 28057360]
10. Reich K, Armstrong AW, Foley P, Song M, Wasfi Y, Randazzo B, et al. Efficacy and safety of guselkumab, an anti-interleukin-23 monoclonal antibody, compared with adalimumab for the treatment of patients with moderate to severe psoriasis with randomized withdrawal and retreatment: results from the phase III, double-blind, placebo- and active comparator controlled VOYAGE 2 trial. Journal of the American Academy of Dermatology 2017;76(3):418-31. [CENTRAL: CN-01341399] [PMID: 28057361]

**Apremilast**

1. Papp K, Reich K, Leonardi CL, Kircik L, Chimenti S, Langley RG, et al. Apremilast, an oral phosphodiesterase 4 (PDE4) inhibitor, in patients with moderate to severe plaque psoriasis: results of a phase III, randomized, controlled trial (Efficacy and Safety Trial Evaluating the Effects of Apremilast in Psoriasis [ESTEEM] 1). Journal of the American Academy of Dermatology 2015;73(1):37-49. [CENTRAL: CN-01085116] [PMID: 26089047]
2. Paul C, Cather J, Gooderham M, Poulin Y, Mrowietz U, Ferrandiz C, et al. Efficacy and safety of apremilast, an oral phosphodiesterase 4 inhibitor, in patients with moderate-to-severe plaque psoriasis over 52 weeks: a phase III, randomized controlled trial (ESTEEM 2). British Journal of Dermatology 2015;173(6):1387-99. [CENTRAL: CN-01133855] [PMID: 26357944]
3. Ohtsuki M, Okubo Y, Komine M, Imafuku S, Day RM, Chen P, et al. Apremilast, an oral phosphodiesterase 4 inhibitor, in the treatment of Japanese patients with moderate to severe plaque psoriasis: efficacy, safety, and tolerability results from a phase 2b randomized controlled trial. Journal of Dermatology 2017;44(8):873-84. [CENTRAL: CN-01600552]
4. Papp K, Cather JC, Rosoph L, Sofen H, Langley RG, Matheson RT, et al. Efficacy of apremilast in the treatment of moderate to severe psoriasis: a randomised controlled trial. Lancet 2012;380(9843):738-46. [CENTRAL: CN-00859723] [PMID: 22748702]
5. Papp KA, Kaufmann R, Thaçi D, Hu C, Sutherland D, Rohane P. Efficacy and safety of apremilast in subjects with moderate to severe plaque psoriasis: results from a phase II, multicenter, randomized, double-blind, placebo-controlled, parallel-group, dose-comparison study. Journal of the European Academy of Dermatology and Venereology 2013;27(3):e376-83. [CENTRAL: CN-01124587] [PMID: 23030767]
6. Van Voorhees AS, Stein Gold L, Lebwohl M, Strober B, Lynde C, Tyring S, et al. Efficacy and safety of apremilast in patients with moderate to severe plaque psoriasis of the scalp: results of a phase 3b, multicenter, randomized, placebo-controlled, doubleblind study. Journal of the American Academy of Dermatology 2020;83(1):96-103.
7. Reich K, Gooderham M, Green L, Bewley A, Zhang Z, Khanskaya I, et al. The efficacy and safety of apremilast, etanercept, and placebo, in patients with moderate to severe plaque psoriasis: 52-week results from a phase 3b, randomized, placebo-controlled trial (LIBERATE). Journal of the European Academy of Dermatology and Venereology 2017;31(3):507-17. [CENTRAL: CN-01285623] [PMID: 27768242]

**Bimekizumab**

1. Papp KA, Merola JF, Gottlieb AB, Griffiths CE, Cross N, Peterson L, et al. Dual neutralization of both interleukin 17A and interleukin 17F with bimekizumab in patients with psoriasis: results from BE ABLE 1, a 12-week randomized, double-blinded, placebo-controlled phase 2b trial. Journal of the American Academy of Dermatology 2018;79(2):277-86.e10. [CENTRAL: CN-01665198]
2. Gordon KB, Foley P, Krueger JG, Pinter A, Reich K, Vender R, et al. Bimekizumab efficacy and safety in moderate to severe plaque psoriasis (BE READY): a multicentre, double-blind, placebo-controlled, randomised withdrawal phase 3 trial. Lancet 2021;397(10273):475-86 Erratum in: Lancet 2021; 397(10288); 1182. [PMID: 33549192]
3. Reich K, Papp KA, Blauvelt A, Langley RG, Armstrong A, Warren RB, et al. Bimekizumab versus ustekinumab for the treatment of moderate to severe plaque psoriasis (BE VIVID): efficacy and safety from a 52-week, multicentre, double-blind, active comparator and placebo controlled phase 3 trial. Lancet 2021;397(10273):487-98.

**Brodalumab**

1. Papp KA, Reich K, Paul C, Blauvelt A, Baran W, Bolduc C, et al. A prospective phase III, randomized,double-blind, placebo-controlled study of brodalumab in patients with moderate-to-severe plaque psoriasis. British Journal of Dermatology 2016;175(2):273-86. [CENTRAL: CN-01208651]
2. Nakagawa H, Niiro H, Ootaki K, Japanese Brodalumab Study Group. Brodalumab, a human anti-interleukin-17-receptor antibody in the treatment of Japanese patients with moderate-to-severe plaque psoriasis: efficacy and safety results from a phase II randomized controlled study. Journal of Dermatological Science 2016;81(1):44-52. [CENTRAL: CN-01133729] [PMID: 26547109]
3. Papp KA, Leonardi C, Menter A, Ortonne JP, Krueger JG, Kricorian G, et al. Brodalumab, an anti-interleukin-17-receptor antibody for psoriasis. New England Journal of Medicine 2012;366(13):1181-9. [CENTRAL: CN-00814009] [PMID: 22455412]
4. Seo SJ, Shin BS, Lee J-H, Jeong H. Efficacy and safety of brodalumab in the Korean population for the treatment of moderate to severe plaque psoriasis: a randomized, phase III, double-blind, placebo-controlled study. Journal of Dermatology 2021;48(6):807-17.
5. Lebwohl M, Strober B, Menter A, Gordon K, Weglowska J, Puig L, et al. Phase 3 studies comparing brodalumab with ustekinumab in psoriasis. New England Journal of Medicine 2015;373(14):1318-28. [CENTRAL: CN-01089800] [PMID: 26422722]

**Certolizumab pegol**

1. Lebwohl M, Blauvelt A, Paul C, Sofen H, Weglowska J, Piguet V, et al. Certolizumab pegol for the treatment of chronic plaque psoriasis: results through 48 weeks of a phase 3, multicenter, randomized, double-blind, etanercept- and placebo-controlled study (CIMPACT). Journal of the American Academy of Dermatology 2018;79(2):266-76.e5. [CENTRAL: CN-01665155]
2. Gottlieb AB, Blauvelt A, Thaçi D, Leonardi CL, Poulin Y, Drew J, et al. Certolizumab pegol for the treatment of chronic plaque psoriasis: results through 48 weeks from 2 phase 3, multicenter, randomized, double-blinded, placebo-controlled studies (CIMPASI-1 and CIMPASI-2). Journal of the American Academy of Dermatology 2018;79(2):302-14.e6. [CENTRAL: CN-01665156]
3. Reich K, Ortonne JP, Gottlieb AB, Terpstra IJ, Coteur G, Tasset C, et al. Successful treatment of moderate to severe plaque psoriasis with the PEGylated Fab' certolizumab pegol: results of a phase II randomized, placebo-controlled trial with a re-treatment extension. British Journal of Dermatology 2012;167(1):180-90. [CENTRAL: CN-00856435] [PMID: 22413944]
4. Umezawa Y, Sakurai S, Hoshii N, Nakagawa H, Group PS Study. Certolizumab pegol for the treatment of moderate to severe plaque psoriasis: 16-week results from a phase 2/3 Japanese study. Dermatology and Therapy 2021;19:19.

**Cyclosporin**

1. Ellis CN, Fradin MS, Messana JM, Brown MD, Siegel MT, Hartley AH, et al. Cyclosporine for plaque-type psoriasis. Results of a multidose, double-blind trial. New England Journal of Medicine 1991;324(5):277-84. [CENTRAL: CN-00072304] [PMID: 1986287]
2. Meffert H, Bräutigam M, Färber L, Weidinger G. Low-dose (1.25 mg/kg) cyclosporin A: treatment of psoriasis and investigation of the influence on lipid profile. Acta Dermato-Venereologica 1997;77(2):137-41. [CENTRAL: CN-00138820] [PMID: 9111826]

**Deucravacitinib**

1. Papp K, Gordon K, Thaçi D, Morita A, Gooderham M, Foley P, et al. Phase 2 trial of selective tyrosine kinase 2 inhibition in psoriasis. New England Journal of Medicine 2018;379(14):1313-21. [CENTRAL: CN-01652897]

**Etanercept**

1. Bachelez H, Van de Kerkhof PC, Strohal R, Kubanov A, Valenzuela F, Lee JH, et al. Tofacitinib versus etanercept or placebo in moderate-to-severe chronic plaque psoriasis: a phase 3 randomised non-inferiority trial. Lancet 2015;386(9993):552-61. [CENTRAL: CN-01091031] [PMID: 26051365]
2. Bagel J, Lynde C, Tyring S, Kricorian G, Shi Y, Klekotka P. Moderate to severe plaque psoriasis with scalp involvement: a randomized, double-blind, placebo-controlled study of etanercept. Journal of the American Academy of Dermatology 2012;67(1):86-92. [CENTRAL: CN-00870940] [PMID: 22014541]
3. Lebwohl M, Blauvelt A, Paul C, Sofen H, Weglowska J, Piguet V, et al. Certolizumab pegol for the treatment of chronic plaque psoriasis: results through 48 weeks of a phase 3, multicenter, randomized, double-blind, etanercept- and placebo-controlled study (CIMPACT). Journal of the American Academy of Dermatology 2018;79(2):266-76.e5. [CENTRAL: CN-01665155]
4. Gottlieb AB, Matheson RT, Lowe N, Krueger GG, Kang S, Gofe BS, et al. A randomized trial of etanercept as monotherapy for psoriasis. Archives of Dermatology 2003;139(12):1627-32. [CENTRAL: CN-00459604] [PMID: 14676082]
5. Gottlieb AB, Leonardi C, Kerdel F, Mehlis S, Olds M, Williams DA. Efficacy and safety of briakinumab vs. etanercept and placebo in patients with moderate to severe chronic plaque psoriasis. British Journal of Dermatology 2011;165(3):652-60. [CENTRAL: CN-00811739] [PMID: 21574983]
6. Reich K, Gooderham M, Green L, Bewley A, Zhang Z, Khanskaya I, et al. The efficacy and safety of apremilast, etanercept, and placebo, in patients with moderate to severe plaque psoriasis: 52-week results from a phase 3b, randomized, placebo-controlled trial (LIBERATE). Journal of the European Academy of Dermatology and Venereology 2017;31(3):507-17. [CENTRAL: CN-01285623] [PMID: 27768242]
7. Reich K, Papp KA, Blauvelt A, Tyring SK, Sinclair R, Thaçi D, et al. Tildrakizumab versus placebo or etanercept for chronic plaque psoriasis (reSURFACE 1 and reSURFACE 2): results from two randomised controlled, phase 3 trials. Lancet 2017;390(10091):276-88. [CENTRAL: CN-01422560]
8. Strober BE, Crowley JJ, Yamauchi PS, Olds M, Williams DA. Efficacy and safety results from a phase III, randomized controlled trial comparing the safety and efficacy of briakinumab with etanercept and placebo in patients with moderate to severe chronic plaque psoriasis. British Journal of Dermatology 2011;165(3):661-8. [CENTRAL: CN-00811738] [PMID: 21574984]
9. Tyring S, Gottlieb A, Papp K, Gordon K, Leonardi C, Wang A, et al. Etanercept and clinical outcomes, fatigue, and depression in psoriasis: double-blind placebo-controlled randomised phase III trial. Lancet 2006;367(9504):29-35. [CENTRAL: CN-00532672] [PMID: 16399150]
10. Griffiths CE, Reich K, Lebwohl M, Van de Kerkhof P, Paul C, Menter A, et al. Comparison of ixekizumab with etanercept or placebo in moderate-to-severe psoriasis (UNCOVER-2 and UNCOVER-3): results from two phase 3 randomised trials. Lancet 2015;386(9993):541-51. [CENTRAL: CN-01091029] [PMID: 26072109]
11. Van de Kerkhof PC, Segaert S, Lahfa M, Luger TA, Karolyi Z, Kaszuba A, et al. Once weekly administration of etanercept 50 mg is efficacious and well tolerated in patients with moderate-to-severe plaque psoriasis: a randomized controlled trial with open-label extension. British Journal of Dermatology 2008;159(5):1177-85. [CENTRAL: CN-00681015] [PMID: 18673365]

**Fumaric acid esters**

1. Mrowietz U, Szepietowski JC, Loewe R, Van de Kerkhof P, Lamarca R, Ocker WG, et al. Efficacy and safety of LAS41008 (dimethyl fumarate) in adults with moderate-to-severe chronic plaque psoriasis: a randomized, double-blind, Fumaderm® - and placebo-controlled trial (BRIDGE). British Journal of Dermatology 2017;176(3):615-23 Corrigendum to: British Journal of Dermatology 2018; 178(1); 308. [CENTRAL: CN-01336917] [PMID: 27515097]
2. Nugteren-Huying WM, Van der SchroeN JG, Hermans J, Suurmond D. Fumaric acid therapy for psoriasis: a randomized, double-blind, placebo-controlled study. Journal of the American Academy of Dermatology 1990;22(2 Pt 1):311-2. [CENTRAL: CN-00066354]
3. NCT03421197. A study to assess the efficacy and safety of PPC-06 (tepilamide fumarate). clinicaltrials.gov/show/ nct03421197 (first received 5 February 2018).

**Guselkumab**

1. Gordon KB, Dufin KC, Bissonnette R, Prinz JC, Wasfi Y, Li S, et al. A phase 2 trial of guselkumab versus adalimumab for plaque psoriasis. New England Journal of Medicine 2015;373(2):136-44. [CENTRAL: CN-01076768] [PMID: 26154787]
2. Ohtsuki M, Kubo H, Morishima H, Goto R, Zheng R, Nakagawa H. Guselkumab, an anti-interleukin-23 monoclonal antibody, for the treatment of moderate to severe plaque type psoriasis in Japanese patients: efficacy and safety results from a phase 3, randomized, double-blind, placebo-controlled study. Journal of Dermatology 2018;45(9):1053-62. [CENTRAL: CN-01646020]
3. Ferris LK, Ott E, Jiang J, Hong HC, Li S, Han C, et al. Efficacy and safety of guselkumab, administered with a novel patient controlled injector (One-Press), for moderate-to-severe psoriasis: results from the phase 3 ORION study. Journal of Dermatological Treatment 2020;31(2):152-9.
4. Blauvelt A, Papp KA, Griffiths CE, Randazzo B, Wasfi Y, Shen Y-K, et al. ENicacy and safety of guselkumab, an anti-interleukin-23 monoclonal antibody, compared with adalimumab for the continuous treatment of patients with moderate to severe psoriasis: results from the phase III, double blinded, placebo- and active comparator-controlled VOYAGE 1 trial. Journal of the American Academy of Dermatology 2016;76(3):405-17. [CENTRAL: CN-01341398] [PMID: 28057360]
5. Reich K, Armstrong AW, Foley P, Song M, Wasfi Y, Randazzo B, et al. Efficacy and safety of guselkumab, an anti-interleukin-23 monoclonal antibody, compared with adalimumab for the treatment of patients with moderate to severe psoriasis with randomized withdrawal and retreatment: results from the phase III, double-blind, placebo- and active comparator controlled VOYAGE 2 trial. Journal of the American Academy of Dermatology 2017;76(3):418-31. [CENTRAL: CN-01341399] [PMID: 28057361]

**Infliximab**

1. Chaudhari U, Romano P, Mulcahy LD, Dooley LT, Baker DG, Gottlieb AB. Efficacy and safety of infliximab monotherapy for plaque-type psoriasis: a randomised trial. Lancet 2001;357(9271):1842-7. [CENTRAL: CN-00348743] [PMID: 11410193]
2. Reich K, Nestle FO, Papp K, Ortonne JP, Evans R, Guzzo C, et al. Infliximab induction and maintenance therapy for moderate-to-severe psoriasis: a phase III, multicentre, double-blind trial. Lancet 2005;366(9494):1367-74. [CENTRAL: CN-00531178] [PMID: 16226614]
3. Menter A, Feldman SR, Weinstein GD, Papp K, Evans R, Guzzo C, et al. A randomized comparison of continuous vs. intermittent infliximab maintenance regimens over 1 year in the treatment of moderate-to-severe plaque psoriasis. Journal of the American Academy of Dermatology 2007;56(1):31.e1-15. [CENTRAL: CN-00576883] [PMID: 17097378]
4. Gottlieb AB, Evans R, Li S, Dooley LT, Guzzo CA, Baker D, et al. Infliximab induction therapy for patients with severe plaque type psoriasis: a randomized, double-blind, placebo-controlled trial. Journal of the American Academy of Dermatology 2004;51(4):534-42. [CENTRAL: CN-00501751] [PMID: 15389187]
5. Torii H, Nakagawa H, Japanese Infliximab Study investigators. Infliximab monotherapy in Japanese patients with moderateto-severe plaque psoriasis and psoriatic arthritis. A randomized, double-blind, placebo-controlled multicenter trial. Journal of Dermatological Science 2010;59(1):40-9. [CENTRAL: CN-00760986] [PMID: 20547039]
6. Yang HZ, Wang K, Jin HZ, Gao TW, Xiao SX, Xu JH, et al. Infliximab monotherapy for Chinese patients with moderate to severe plaque psoriasis: a randomized, double-blind, placebo-controlled multicenter trial. Chinese Medical Journal 2012;125(11):1845-51. [CENTRAL: CN-00904898] [PMID: 22884040]

**Ixekizumab**

1. Leonardi C, Matheson R, Zachariae C, Cameron G, Li L, Edson Heredia E, et al. Anti-interleukin-17 monoclonal antibody ixekizumab in chronic plaque psoriasis. New England Journal of Medicine 2012;366(13):1190-9. [CENTRAL: CN-00814008] [PMID: 22455413]
2. NCT03364309. A study of ixekizumab (LY2439821) in Chinese participants with moderate-to-severe plaque psoriasis. clinicaltrials.gov/show/nct03364309 (first received 6 December 2017).
3. Gordon KB, Blauvelt A, Papp KA, Langley RG, Luger T, Ohtsuki M, et al. Phase 3 trials of ixekizumab in moderate-to-severe plaque psoriasis. New England Journal of Medicine 2016;375(4):345-56. [CENTRAL: CN-01167902] [PMID: 27299809]

**Methotrexate**

1. Hunter GA, Turner AN. Methotrexate in the treatment of psoriasis: a controlled clinical trial. Australasian Journal of Dermatology 1963;7(2):91-2. [CENTRAL: CN-01437408] [PMID: 14148789]
2. Warren RB, Mrowietz U, Von Kiedrowski R, Niesmann J, Wilsmann-Theis D, Ghoreschi K, et al. An intensified dosing schedule of subcutaneous methotrexate in patients with moderate to severe plaque-type psoriasis (METOP): a 52 week, multicentre, randomised, double-blind, placebo-controlled, phase 3 trial. Lancet 2017;389(10068):528-37. [CENTRAL: CN-01330160] [PMID: 28012564
3. Saurat JH, Stingl G, Dubertret L, Papp K, Langley RG, Ortonne JP, et al. Efficacy and safety results from the randomized controlled comparative study of adalimumab vs. methotrexate vs. placebo in patients with psoriasis (CHAMPION). British Journal of Dermatology 2008;158(3):558-66. [CENTRAL: CN-00628565] [PMID: 18047523]

**Netakimab**

1. NCT02762994. International clinical trial to evaluate efficacy and safety of multiple subcutaneous injections of BCD-085 in various doses in patients with moderate to severe plaque psoriasis. clinicaltrials.gov/show/nct02762994 (first received 5 May 2016).
2. Puig L, Bakulev AL, Kokhan MM, Samtsov AV, Khairutdinov VR, Morozova MA, et al. Efficacy and safety of netakimab, a novel anti-IL-17 monoclonal antibody, in patients with moderate to severe plaque psoriasis. Results of a 54-week randomized double-blind placebo-controlled PLANETA clinical trial. Dermatology and Therapy 2021;11(4):1319-32.

**Secukinumab**

1. Sigurgeirsson B, Schäkel K, Hong CH, Effendy I, Placek W, Rich P, et al. Efficacy, tolerability, patient usability, and satisfaction with a 2 mL pre-filled syringe containing secukinumab 300 mg in patients with moderate to severe plaque psoriasis: results from the phase 3 randomized, double-blind, placebo-controlled ALLURE study. Journal of Dermatological Treatment 2021 Apr 26 [Epub ahead of print]. [DOI: 10.1080/09546634.2021.1902925]
2. Cai L, Zhang JZ, Yao X, Gu J, Liu Q-Z, Zheng M, et al. Secukinumab demonstrates high efficacy and a favorable safety profile over 52 weeks in Chinese patients with moderate to severe plaque psoriasis. Chinese Medical Journal 2020;133(22):2665-73.
3. Von Stebut E, Reich K, Thaçi D, Koenig W, Pinter A, Korber A, et al. Impact of secukinumab on endothelial dysfunction and other cardiovascular disease parameters in psoriasis patients over 52 weeks. Journal of Investigative Dermatology 2019;139(5):1054-62.
4. Blauvelt A, Prinz JC, Gottlieb AB, Kingo K, Sofen H, RuerMulard M, et al. Secukinumab administration by pre-filled syringe: efficacy, safety and usability results from a randomized controlled trial in psoriasis (FEATURE). British Journal of Dermatology 2015;172(2):484-93. [CENTRAL: CN-01052626] [PMID: 25132411]
5. Langley RG, Elewski BE, Lebwohl M, Reich K, Griffiths CE, Papp K, et al. Secukinumab in plaque psoriasis - results of two phase 3 trials. New England Journal of Medicine 2014;371(4):326-38. [CENTRAL: CN-00999505] [PMID: 25007392]
6. Paul C, Lacour JP, Tedremets L, Kreutzer K, Jazayeri S, Adams S, et al. Efficacy, safety and usability of secukinumab administration by autoinjector/pen in psoriasis: a randomized, controlled trial (JUNCTURE). Journal of the European Academy of Dermatology and Venereology 2015;29(6):1082-90. [CENTRAL: CN-01043227] [PMID: 25243910]
7. NCT03055494. Study to explore the effect of secukinumab, compared to placebo, on fat tissue and skin in plaque psoriasis patients (ObePso-S). clinicaltrials.gov/show/nct03055494 (first received 16 February 2017).
8. NCT03535194. A study to assess if mirikizumab is effective and safe compared to secukinumab and placebo in moderate to severe plaque psoriasis (OASIS-2). clinicaltrials.gov/show/ nct03535194 (first received 24 May 2018).
9. NCT03589885. Study of efficacy and safety of secukinumab 2 mL auto-injector (300 mg) in subjects with moderate to severe plaque psoriasis (MATURE). clinicaltrials.gov/show/nct03589885 (first received 18 July 2018).
10. Papp KA, Langley RG, Sigurgeirsson B, Abe M, Baker DR, Konno P, et al. Efficacy and safety of secukinumab in the treatment of moderate-to-severe plaque psoriasis: a randomized, double-blind, placebo-controlled phase II dose-ranging study. British Journal of Dermatology 2013;168(2):412-21. [CENTRAL: CN-00967073] [PMID: 23106107]
11. Reich K, Papp KA, Matheson RT, Tu JH, Bissonnette R, Bourcier M, et al. Evidence that a neutrophil-keratinocyte crosstalk is an early target of IL-17A inhibition in psoriasis. Experimental Dermatology 2015;24(7):529-35. [CENTRAL: CN-01171151] [PMID: 25828362]
12. Rich P, Sigurgeirsson B, Thaçi D, Ortonne JP, Paul C, Schopf RE, et al. Secukinumab induction and maintenance therapy in moderate-to-severe plaque psoriasis: a randomized, double-blind, placebo-controlled, phase II regimen-finding study. British Journal of Dermatology 2013;168(2):402-11. [CENTRAL: CN-00965685] [PMID: 23362969]
13. Reich K, Sullivan J, Arenberger P, Mrowietz U, Jazayeri S, Augustin M, et al. Secukinumab shows significant efficacy in nail psoriasis: week 32 results from the TRANSFIGURE study. Annals of the Rheumatic Diseases 2016;75(Suppl 2):603-4. [CENTRAL: CN-01761193]
14. Gelfand JM, Shin DB, DuNin KC, Armstrong AW, Blauvelt A, Tyring SK, et al. A randomized placebo-controlled trial of secukinumab on aortic vascular inflammation in moderate to severe plaque psoriasis (VIP-S). Journal of Investigative Dermatology 2020;140(9):1784-93.e2.

**Risankizumab**

1. Blauvelt A, Leonardi CL, Gooderham M, Papp KA, Philipp S, Wu JJ, et al. Efficacy and safety of continuous risankizumab therapy vs treatment withdrawal in patients with moderate to severe plaque psoriasis: a phase 3 randomized clinical trial. JAMA Dermatology 2020;156(6):649-58.
2. Blauvelt A, Papp KA, Gooderham M, Langley RG, Leonardi C, Lacour JP, et al. Efficacy and safety of risankizumab, an interleukin-23 inhibitor, in patients with moderate-to-severe chronic plaque psoriasis: 16-week results from the phase III IMMhance trial. British Journal of Dermatology 2017;177(5):e248. [CENTRAL: CN-01452512]
3. Ohtsuki M, Fujita H, Watanabe M, Suzaki K, Flack M, Huang X, et al. Efficacy and safety of risankizumab in Japanese patients with moderate to severe plaque psoriasis: results from the SustaIMM phase 2/3 trial. Journal of Dermatology 2019;46(8):686-94
4. Gordon KB, Strober B, Lebwohl M, Augustin M, Blauvelt A, Poulin Y, et al. ENicacy and safety of risankizumab in moderate-to-severe plaque psoriasis (UltIMMa-1 and UltIMMa-2): results from two double-blind, randomised, placebo-controlled and ustekinumab-controlled phase 3 trials. Lancet 2018;392(10148):650-61. [CENTRAL: CN-01649259]

**Sonelokimab**

1. Papp KA, Weinberg MA, Morris A, Reich K. IL17A/F nanobody sonelokimab in patients with plaque psoriasis: a multicentre, randomised, placebo-controlled, phase 2b study. Lancet 2021;397(10284):1564-75 Erratum in Lancet 2021; 397(10290):2150.

**Tildrakizumab**

1. Papp K, Thaçi D, Reich K, Riedl E, Langley RG, Krueger JG, et al. Tildrakizumab (MK-3222), an anti-interleukin-23p19 monoclonal antibody, improves psoriasis in a phase IIb randomized placebo-controlled trial. British Journal of Dermatology 2015;173(4):930-9. [CENTRAL: CN-01105188] [PMID: 26042589]
2. Reich K, Papp KA, Blauvelt A, Tyring SK, Sinclair R, Thaçi D, et al. Tildrakizumab versus placebo or etanercept for chronic plaque psoriasis (reSURFACE 1 and reSURFACE 2): results from two randomised controlled, phase 3 trials. Lancet 2017;390(10091):276-88. [CENTRAL: CN-01422560]

**Ustekinumab**

1. Lebwohl M, Strober B, Menter A, Gordon K, Weglowska J, Puig L, et al. Phase 3 studies comparing brodalumab with ustekinumab in psoriasis. New England Journal of Medicine 2015;373(14):1318-28. [CENTRAL: CN-01089800] [PMID: 26422722]
2. Reich K, Papp KA, Blauvelt A, Langley RG, Armstrong A, Warren RB, et al. Bimekizumab versus ustekinumab for the treatment of moderate to severe plaque psoriasis (BE VIVID): efficacy and safety from a 52-week, multicentre, double-blind, active comparator and placebo controlled phase 3 trial. Lancet 2021;397(10273):487-98.
3. Igarashi A, Kato T, Kato M, Song M, Nakagawa H, Japanese Ustekinumab Study Group. Efficacy and safety of ustekinumab in Japanese patients with moderate-to-severe plaque type psoriasis: long-term results from a phase 2/3 clinical trial. Journal of Dermatology 2012;39(3):242-52. [CENTRAL: CN-00860708] [PMID: 21955098]
4. Krueger GG, Langley RG, Leonardi C, Yeilding N, Guzzo C, Wang Y, et al. A human interleukin-12/23 monoclonal antibody for the treatment of psoriasis. New England Journal of Medicine 2007;356(6):580-92. [CENTRAL: CN-00575216] [PMID: 17287478]
5. Zhu X, Zheng M, Song M, Shen YK, Chan D, Szapary PO, et al. Efficacy and safety of ustekinumab in Chinese patients with moderate to severe plaque-type psoriasis: results from a phase 3 clinical trial (LOTUS). Journal of Drugs in Dermatology 2013;12(2):166-74. [CENTRAL: CN-00965604] [PMID: 23377389]
6. Tsai TF, Ho JC, Song M, Szapary P, Guzzo C, Shen YK, et al. Efficacy and safety of ustekinumab for the treatment of moderate-to-severe psoriasis: a phase III, randomized, placebo controlled trial in Taiwanese and Korean patients (PEARL). Journal of Dermatological Science 2011;63(3):154-63. [CENTRAL: CN-00810821] [PMID: 21741220]
7. Leonardi CL, Kimball AB, Papp KA, Yeilding N, Guzzo C, Wang Y, et al. Efficacy and safety of ustekinumab, a human interleukin-12/23 monoclonal antibody, in patients with psoriasis: 76-week results from a randomised, double-blind, placebo-controlled trial (PHOENIX 1). Lancet 2008;371(9625):1665-74. [CENTRAL: CN-00631485] [18486739]
8. Papp KA, Langley RG, Lebwohl M, Krueger GG, Szapary P, Yeilding N, et al. Efficacy and safety of ustekinumab, a human interleukin-12/23 monoclonal antibody, in patients with psoriasis: 52-week results from a randomised, double-blind, placebo-controlled trial (PHOENIX 2). Lancet 2008;371(9625):1675-84. [CENTRAL: CN-00631486] [PMID: 18486740]
9. Gordon KB, Strober B, Lebwohl M, Augustin M, Blauvelt A, Poulin Y, et al. Efficacy and safety of risankizumab in moderate-to-severe plaque psoriasis (UltIMMa-1 and UltIMMa-2): results from two double-blind, randomised, placebo controlled and ustekinumab-controlled phase 3 trials. Lancet 2018;392(10148):650-61. [CENTRAL: CN-01649259]
10. Gelfand JM, Shin DB, Alavi A, Torigian DA, Werner T, Papadopoulos M, et al. A phase IV, randomized, doubleblind, placebo-controlled crossover study of the effects of ustekinumab on vascular inflammation in psoriasis (the VIP-U Trial). Journal of Investigative Dermatology 2020;140(1):85-93.e2

**Studies Excluded Due to Inability to Find Primary Data (all acitretin versus placebo)**

1. Goldfarb MT, Ellis CN, Gupta AK, Tincoff T, Hamilton TA, Voorhees JJ. Acitretin improves psoriasis in a dose-dependent fashion. Journal of the American Academy of Dermatology 1988;18(4 Pt 1):655-62. [CENTRAL: CN-00053926] [PMID: 2967310]
2. Saurat JH, Geiger JM, Amblard P, Beani JC, Boulanger A, Claudy A, et al. Randomized double-blind multicenter study comparing acitretin-PUVA, etritinate-PUVA and placeboPUVA in the treatment of severe psoriasis. Dermatologica 1988;177(4):218-24. [CENTRAL: CN-00058056] [PMID: 2976000]
3. Sommerburg C, Kietzmann H, Eichelberg D, Goos M, Heese A, Holzle E, et al. Acitretin in combination with PUVA: a randomized double-blind placebo-controlled study in severe psoriasis. Journal of the European Academy of Dermatology and Venereology 1993;2(4):308-17. [CENTRAL: CN-00180920] [EMBASE: 1993350796]
4. Yilmaz E, Yilmaz F, Yerebakan O. Re-PUVA therapy for psoriasis vulgaris: an effective choice. Journal of the European Academy of Dermatology and Venereology 2002;16(Suppl S1):258. [CENTRAL: CN-00416979]
